# Association of applicant demographic factors with medical school acceptance

**DOI:** 10.1186/s12909-023-04897-8

**Published:** 2023-12-14

**Authors:** Michael A. Perez, Cheyenne Williams, Korey Henderson, Ryan McGregor, Neha Vapiwala, Judy A. Shea, C. Jessica Dine

**Affiliations:** 1 Department of Neurology, Hospital of the University of Pennsylvania, Philadelphia, PA US; 2 Department of Urology, Hospital of the University of Pennsylvania, Philadelphia, PA US; 3 Perelman School of Medicine, University of Pennsylvania, Philadelphia, PA US; 4 Department of Radiation Oncology, Hospital of the University of Pennsylvania, Philadelphia, PA US; 5 Division of General Internal Medicine, Perelman School of Medicine, University of Pennsylvania, Philadelphia, PA US; 6 Department of Medicine, Division of Allergy, Pulmonary and Critical Care, Hospital of the University of Pennsylvania, Philadelphia, PA US; 7 Leonard Davis Institute of Economics, University of Pennsylvania, Philadelphia, PA US

**Keywords:** Medical school admission, Low-income applicants, First-generation applicants, Holistic admissions

## Abstract

**Background:**

Medical school acceptance rates in the United States (US) have been lower for applicants who identify as Underrepresented-in-Medicine (UiM) compared to non-UiM applicants. The gap between UiM and no-UiM groups is narrowing in recent years. Less well-studied are associations of acceptance decisions with family income and parental education. This study’s purpose is to evaluate the relationships between medical school acceptance and family income, parental education status, racial/ethnic background, Grade Point Average (GPA), Medical College Admission Test (MCAT) score, and participation in extracurricular activities.

**Methods:**

This is a cross-sectional study of first-time US medical school applicants between 2017 and 2020. Acceptance rates for first-time applicants were calculated for first-generation (FG), low-income (LI), and UiM applicants. Associations of these attributes with MCAT scores, science GPAs, and seven categories of extracurricular activities were evaluated. Regression analyses estimated associations between acceptance to medical school with all variables with and without interaction terms (FG*URM, LI*URM, FG*LI).

**Results:**

The overall acceptance rate for first-time applicants from 2017–2020 was 45.3%. The acceptance rates among FG, LI and UiM applicants were 37.9%, 39.6% and 44.2%, respectively. In univariable logistic regression analyses, acceptance was negatively associated with being FG (OR: 0.68, CI: 0.67–0.70), LI (OR: 0.70, CI: 0.69–0.72), and UiM (OR: 0.95, CI: 0.93–0.97). In multivariable regression, acceptance was most strongly associated with science GPA (OR: 7.15, CI: 6.78–7.54 for the highest quintile) and UiM (OR: 5.56, CI: 5.48–5.93) status and MCAT score (OR: 1.19, CI: 1.18–1.19), FG (OR: 1.14, CI: 1.10–1.18), and most extracurricular activities. Including interaction terms revealed a negative association between acceptance and LI (OR:0.90, CI: 0.87–0.94) and FG was no longer significant (OR:1.10, CI:0.96–1.08).

**Conclusions:**

Collectively these results suggest medical school admissions committees may be relying on holistic admission practices. While MCAT and GPA scores continue to predict acceptance, individuals from racially and ethnically UiM backgrounds have favorable odds of acceptance when controlling for MCAT and GPA. However, these positive associations were not seen for low-income and first-generation applicants. Additional preparation for college and the MCAT for these latter groups may help further diversify the medical profession.

**Supplementary Information:**

The online version contains supplementary material available at 10.1186/s12909-023-04897-8.

## Background

On average over 50,000 aspiring physicians apply each year to United States (US) allopathic medical schools. However, only around 40–45% of these applicants obtain an acceptance [1]. Importantly, medical school matriculants have rarely reflected the US population in terms of race and ethnicity. Individuals from racial and ethnic minority groups have historically been Underrepresented in Medicine (UiM) with respect to medical school enrollment compared to the general population [2]. Stark disparities have also been noted in socioeconomic backgrounds of matriculants. Since 1987, half of entering US medical students come from families in the highest income quintile, while only 5.5% of entering US medical students come from families in the lowest income quintile [3, 4].

Many calls have been made to address underrepresentation in medical schools. For example, the 2009 Liaison Committee for Medical Education (LCME) accreditation guidelines required medical schools to “make admission to medical education more accessible to potential applicants of diverse backgrounds” [5]. In 2012, the American Association of Medical Colleges (AAMC) introduced a socioeconomic indicator to provide admission committees a way to identify socioeconomically disadvantaged applicants [6]. In 2016, the AAMC recommended that medical school admissions committees practice “holistic admissions” [7]. The hope is that using a more holistic approach will counteract the racial and socioeconomic disparities observed in MCAT scores and GPA averages that may be secondary to structural racism [8–11]. Moreover, research has demonstrated that medical student and physician diversity is associated with improved educational experiences as well as patient outcomes [11–14]. Despite this concerted effort to raise awareness of observed disparities, socioeconomic diversity had failed to improve among the medical student population as of 2023 [1, 15].

To date, most descriptions of medical school applicants’ background have focused on one or two background features. The purpose of this study is to examine the association of multiple factors, alone and in combination, on the likelihood of acceptance. Of primary interest are self-identification as a first-generation college student (FG) and familial low-income (LI). UiM status is also included as it is closely intertwined with being FG and LI. We hypothesize that all three demographic factors negatively predict medical school acceptance and are also negatively associated with MCAT scores and science GPA. Furthermore, we explore the relationship between these factors and participation in extracurricular activities. Additionally, we hypothesize that MCAT scores, science GPA, and participation in extracurricular activities are each positively associated with acceptance and mediates the negative impact of the socioeconomic indicators.

## Methods

The research protocol for this study was deemed exempt from review by the institutional review board at the University of Pennsylvania. The data used in this project were originally collected by the American Medical College Application Service (AMCAS) and maintained within the AAMC Applicant Matriculant Data file. Data were obtained from the AAMC through the standard data request process.

### Data sources

De-identified individual applicant-level data from AMCAS were obtained from the AAMC Applicant Matriculant Data File for all medical school applicants with application years between 2014 and 2021. Only first-time applicants were included in these analyses. The following applicant characteristics were extracted: sex, age, highest parental education level, childhood family income, race/ethnicity, Medical College Admissions Test 2015 (MCAT) version scores (defined below), science and non-science grade point average (GPA), the type and number of extracurricular activities, and final acceptance to medical school decision. Apart from MCAT and GPA, all the characteristics were self-reported by each applicant. Notably, the AAMC launched a new version of the MCAT in 2015 (MCAT). Only applicants with scores for the newest MCAT version were included for analysis. Also, applicants in 2021 (the first year of the pandemic) were very different than those in earlier years in terms of number and characteristics as there was an unprecedented 17% increase in total applicants. Thus, the analytic data file was limited to the 2017–2020 applicants.

### Definition of variables

FG status included those applicants without any parents (up to six potentially coded) with a bachelor's degree or higher. LI status was defined as having reported childhood family income of less than $75,000. This value was chosen as it fell under 300% of the poverty level for a family of four in 2019. Furthermore, this value is consistent with the criteria to receive the AMCAS Fee Assistance Program (FAP) for application years within the dataset. Individuals with missing data or who reported “do not know” were excluded from the analyses. UiM included individuals who identified as Black, Hispanic, Pacific Islander/Native Hawaiian, or Native American, within any of their self-selected designations, while applicants who indicated only Asian and/or White race were considered non-UiM. Science GPA was significantly right-skewed and therefore divided into quintiles for analysis. Extracurricular activities were organized into seven dichotomous (0/1) categories: 1) Employment; 2) Medical Employment; 3) Scholarly activities which included publications, conference attendance, and presentations or poster presentations; 4) Honors/Awards; 5) Shadowing; 6) Community Service; and 7) Other activities which included research, teaching, leadership experiences, and any other extra-curricular activities. Most activities were highly skewed and converted to a binary characteristic if the applicant had one or more activities in this category. Normally distributed activities (community service and other activities) were divided at the median.

### Statistical analysis

Within groups defined by FG, LI, and UiM status, analyses summarize overall differences in MCAT averages, GPA averages, the type and number of extracurricular activities, and acceptance rates overall for medical school applicants. To test our hypothesis that applicant characteristics of FG, LI, and UiM have lower MCAT and science GPA while having higher employed activities and lower non-employment activities compared to other applicants, chi-square tests were conducted for each relationship. Univariable and multivariable regression analyses were performed to evaluate the association of FG, LI, UiM, MCAT score (per one point), science GPA quintile, and extracurricular activities with medical school acceptance among applicants. Interaction terms between FG and UiM, LI and UiM, and FG and LI were included in multivariable models to examine stability of results. Variance Inflation Factors (VIF) were calculated between variables to evaluate for evidence of multi-collinearity. Given the large number of analyses performed, statistical significance was set at *p* <  = 0.01. Statistical analyses were performed in SPSS (IBM SPSS Statistics for Windows, version 26, Armonk, NY: IBM Corp).

## Results

During the 2017–2020 time period, AMCAS collected 153,664 first-time applicants to US allopathic medical schools. Of the applicants who reported parent education level or family income, 30,455 (19.8%) were FG college students or graduates and 54,735 (35.6%) were LI (Table 1). The average MCAT score among applicants was 506.00 (SD = 9.54). The average science GPA of the total applicant pool was 3.51 (SD = 0.42). Applicant participation in each of the Extracurricular Activities categories ranged from 37.0% (*n* = 56,820) for Scholarly Activities to 74.3% (*n* = 114,164) for Shadowing (Table 1). All variables in Table 1 had statistically significant differences between the accepted and non-accepted pool except for employment.

**Table 1:** Demographics

	**All Applicants** *N* = 153,664	**Accepted Applicants** *N* = 69,569 (45.3%)	**Not Accepted Applicants** *N* = 84,095 (54.7%)
**Sex, n/%**			
Female	82,190 / 53.5%	37,683 / 54.2%	44,507 / 52.9%
Male	71,356 / 46.4%	31,823 / 45.7%	39,533 / 47.0%
Not Reported	118 / 0.1%	63 / 0.1%	55 / 0.1%
**Age, n/%**			
UNDER 22	10,896 / 7.1%	6,260 / 9.0%	4,636 / 5.5%
22	45,647 / 29.7%	23,851 / 34.3%	21,796 / 25.9%
23	36,610 / 23.8%	17,416 / 25.0%	19,194 / 22.8%
24	23,485 / 15.3%	9,922 / 14.3%	13,563 / 16.1%
25	12,596 / 8.2%	4,553 / 6.5%	8,043 / 9.6%
26	7,422 / 4.8%	2,433 / 3.5%	4,989 / 5.9%
27	4,591 / 3.0%	1,466 / 2.1%	3,125 / 3.7%
OVER 27	12,417 / 8.1%	3,668 / 5.3%	8,749 / 10.4%
**First-Generation College Student, n/%**	30,455 / 19.8%	11,529 / 16.6%*	18,926 / 22.5%*
**Low-income (< $75,000 Childhood Income), n/%**	54,735 / 35.6%	21,702 / 31.2%*	33,033 / 39.3%*
**Underrepresented in Medicine, n/% (95% CI)**	31,587 / 20.6%	13,975 / 10.1%*	17,612 / 20.9%*
**MCAT2015 Total Score, Mean (SD)**	506.00 (9.54)	511.70 (6.46)*	501.46 (9.15)*
**Science GPA, Mean (SD)**	3.51 (0.42)	3.70 (0.28)*	3.35 (0.45)*
Quintile 1 (< 3.19)	28,974 / 18.9%	3,840 / 5.5%	25,134 / 19.9%
Quintile 2 (3.19 – 3.48)	29,495 / 19.2%	9,664 / 14.4%	19,831 / 23.6%
Quintile 3 (3.49 – 3.69)	29,402 / 19.1%	14,219 / 20.4%	15,183 / 18.1%
Quintile 4 (3.70 – 3.87)	29,208 / 19.0%	17,474 / 25.1%	11,734 / 14.0%
Quintile 5 (3.88 – 4.00)	30,617 / 19.9%	22,126 / 31.8%	8,491 / 10.1%
**Non-Science GPA, Mean (SD)**	3.74 (0.27)	3.83 (0.20)*	3.66 (0.30)*
**Extracurricular Activities, n (%)**			
Employment	90,657 / 59.0%	41,129 / 59.1%	49,528 / 58.9%
Medical employment	68,428 / 44.5%	29,214 / 42.0%*	39,214 / 46.6%*
Scholarly activities	56,820 / 37.0%	28,769 / 41.4%*	28,051 / 33.4%*
Honors/awards	78,505 / 51.1%	41,129 / 59.1%*	37,376 / 44.4%*
Shadowing	114,164 / 74.3%	56,053 / 80.6%*	58,111 / 69.1%*
Community service	75,018 / 48.8%	38,634 / 55.5%*	36,384 / 43.3%*
Other activities	69,625 / 45.3%	38,935 / 56.0%*	30,690 / 36.5%*

^*^ *p* < 0.01

**Fig. 1:**
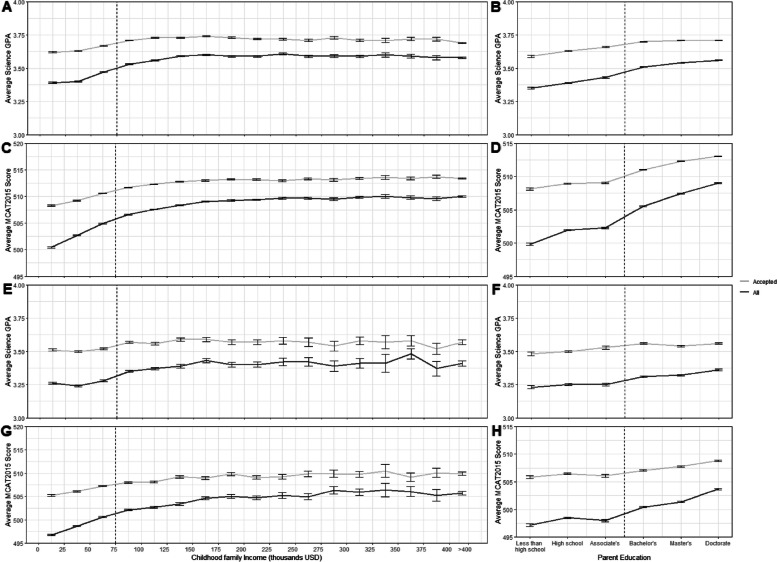
Average GPA and MCAT scores for all applicants versus accepted applicants along with all UiM applicants versus UiM accepted applicants within each income category and parent education level. Average MCAT and GPAs increase in association with increased family income (**a**-**b**) and parent education level (**c**-**d**) for both applicants (dark gray line) and accepted applicants (light gray line). Similar findings were observed for UiM applicants and UiM accepted applicants (**e**–**h**). accepted applicants’ MCAT scores and GPA’s are significantly higher than accepted students’ metrics for every income category and parental education level. Values represent mean ± standard error

As shown in Additional File 1, MCAT and science GPA scores were lower for applicants identified as FG, LI or UiM compared to their non-identified counterparts. Figure 1 shows in detail how FG, LI, and UiM status were negatively related to MCAT and GPA. Self-identified FG, LI, and UiM applicants also participated less frequently in extracurricular activities, except FG and LI were more likely to report medical and non-medical employment. The overall acceptance rates for FG, LI, and UiM applicants were 37.9%, 39.6%, and 44.2%, respectively. The acceptance rate for the non-UiM counterparts was 45.5%.

### First-Generation

The mean MCAT score of FG applicants was significantly lower compared to non-FG applicant pool (502.59 (SD 9.75) vs 506.86 (SD 9.29), *p*-value < 0.01 (Additional File 1). Similarly, the mean science GPA of FG applicants was 3.42 compared to a mean science GPA of 3.53 of the non-FG applicant pool, *p*-value 0.45 (Additional File 1). FG applicants had higher engagement rates in both non-medical and medical employment of 63.4% (19,316/30,455) and 50.4% (15,340/30,455), respectively, compared to the non-FG applicant pool’s rates of 57.9% and 43.1%. FG applicants had lower engagement in honors/awards, shadowing, community service, and other activities but similar rates of involvement in scholarly activities (Additional File 1). However, in many cases the differences were less than 1–2%.

### Low-income

The MCAT and science GPA of LI applicants were significantly lower than the non-LI applicant pool (Additional File 1); 503.55 (SD 1.00) versus 507.38 (SD 8.98) for MCAT and 3.44 versus 3.55 for GPA. LI applicants did have higher engagement in both paid employment categories and scholarly activities, but lower engagement in all other activities such as honors/awards (50.7% versus 51.3%) and shadowing (73.3% versus 74.8%), but again, differences were small (Additional File 1).

### UiM

Finally, mean MCAT score of UiM applicants was 500.21 (SD 10.05) compared to the non-UiM applicant pool’s mean of 507.38 (SD 8.79) with a *p*-value of < 0.01. GPAs were also lower (3.30 versus 3.56, respectively). UiM applicants had lower engagement in all categories of extracurricular activities, including employment.

On univariate logistic regression analysis, acceptance was negatively associated with FG college status (OR: 0.68, CI: 0.67–0.70) and LI status (OR: 0.70, CI: 0.69–0.72) (Table 2). UiM status was only slightly negatively associated (OR: 0.95, CI: 0.93–0.97) whereas higher MCAT scores and science GPA were positively associated with acceptance (Table 2). All extracurricular activities, apart from employment, were positively associated with acceptance (Table 2). Subsequent multivariable analysis demonstrated that medical school acceptance was most strongly associated with science GPA (OR: 7.15, CI: 6.78–7.54 for the highest quintile) and UiM status (OR: 5.58, CI: 5.48–5.93), and positively associated with MCAT score (OR: 1.19, CI: 1.18–1.19) and FG status (OR: 1.14, CI: 1.10–1.18). All extracurricular activities, apart from non-medical employment (OR: 1.01, CI: 0.99–1.04), were also associated with medical school acceptance. Low-income status (OR: 1.00, CI: 0.97–1.03) was not statistically significant. VIF values were obtained for the UiM, FG, and LI and varied between 1.04 and 1.15 which were not suggestive of multi-collinearity. Associations of each variable with medical school acceptance remained the same when adding interaction terms to the multivariate regression (Table 2) model (FG*UiM, LI*UiM, FG*LI) except LI was now also negatively associated with medical school acceptance (OR:0.90, CI: 0.87–0.94) where FG was no longer associated (OR:1.01, CI:0.96–1.08).

**Table 2:** Associations with medical school acceptance

	**Univariate**		**Multivariate**		**Multivariate with Interaction Terms**	
	**OR**	**OR 95% CI**	**OR**	**OR 95% CI**	**OR**	**OR 95% CI**
First-generation (FG)	0.68	0.67–0.70	1.14	1.10–1.18	1.01	0.96–1.08
Low-income (LI)	0.70	0.69–0.72	1.00	0.97–1.03	0.90	0.87–0.94
UiM	0.95	0.93–0.97	5.58	5.48–5.93	5.42	5.13–5.72
First-generation x UiM					0.80	0.74–0.87
Low-income x UiM					1.20	1.11–1.29
First-generation x Low-income					1.34	1.25–1.45
MCAT2015	1.18	1.18–1.18	1.19	1.18–1.19	1.19	1.18–1.19
Science GPA^a^						
Quintile 2 (3.19–3.48)	3.19	3.06–3.32	2.30	2.18–2.42	2.30	2.19–2.42
Quintile 3 (3.49–3.69)	6.13	5.88–6.39	3.84	3.66–4.03	3.84	3.55–4.04
Quintile 4 (3.70–3.87)	9.75	9.35–10.16	5.26	5.00–5.53	5.27	5.00–5.54
Quintile 5 (3.88–4.00)	17.06	16.36–17.76	7.15	6.78–7.54	7.16	6.79–7.55
Employment	1.01	0.99–1.03	1.01	0.99–1.04	1.01	0.96–1.04
Medical employment	0.83	0.81–0.85	1.26	1.22–1.29	1.26	1.22–1.29
Scholarly activities	1.41	1.38–1.44	1.09	1.06–1.12	1.09	1.06–1.11
Honors/awards	1.81	1.77–1.85	1.19	1.16–1.22	1.19	1.16–1.22
Shadowing	1.85	1.81–1.90	1.57	1.52–1.63	1.57	1.52–1.63
Community service	1.64	1.61–1.67	1.47	1.43–1.51	1.47	1.43–1.51
Other activities	2.12	2.17–2.26	1.47	1.43–1.51	1.47	1.43–1.51

^a^Science GPAs were compared to quintile 1

## Discussion

This study demonstrates that the overall acceptance rates for FG, LI, and UiM applicants are lower compared to the non-FG, non-LI, and non-UiM counterparts. However, UiM applicants have only a modest difference in acceptance rates. As hypothesized, each of these applicant factors alone is a negative predictor of acceptance. Moreover, as hypothesized, the mean MCAT and science GPA are positive predictors of acceptance, consistent with prior literature [9, 16]. Additionally, a deeper dive into interrelationships among the predictors showed clear negative relationships between FG, LI, and UiM status and MCAT and science GPA scores. New to the literature is the observation that most categories of extracurricular activities are positive predictors of acceptance. Participation in extracurricular activities was variable within the three self-identified focal groups, but differences were not large except for higher participation in employment for FG and LI applicants. Interestingly, the multivariable results showed that higher MCAT and science GPA scores mediate the initial negative results, especially for UiM applicants.

Thus, what do these results mean in the era of recommended holistic review? The AAMC has officially stated that the application review process should balance the importance of academic metrics with the “context of applicants’ experiences and attributes,” as well as “educational opportunities, lived experiences, [and] academic trajectories” [17]. Surveys to members of medical school admissions committees across the country found that parental education/occupation/socioeconomic status (SES) was deemed to have a medium mean importance rating, along with factors such as non-science GPA and research/lab experiences [16]. However, our multivariable logistic regression analysis including interaction terms found that LI status had a negative association with acceptance while FG college status had no statistically significant association with acceptance. UiM status had a more significant positive association with acceptance. These results suggest that, while US medical schools may be practicing holistic admissions as evident by these analyses, FG or LI college applicants may still struggle with medical school acceptance.

Furthermore, these results demonstrate that applicants with family income less than $75,000 and FG college students are relatively under-accepted. These findings corroborate prior literature that socioeconomically disadvantaged students are underrepresented in the current medical student population [3, 4, 15]. Studies outside the US have also found that lower-income applicants experienced statistically significantly lower acceptance rates to medical school than individuals from privileged socioeconomic backgrounds [18, 19]. Additionally, socioeconomically disadvantaged applicants are more likely to perceive the pre-medical path and medical school application process as daunting and less attainable, potentially resulting in fewer applicants from these backgrounds [20]. However, a key issue is that low-income students within this study, as well as others, have significantly lower performance in the MCAT and GPA metrics, and it is unlikely that parity will be achieved in an admissions process that primarily uses these data to inform decisions. Further considerations for these disparities in acceptance to medical school amongst lower-income applicants includes the possibility that they may not have the funds to engage in MCAT preparatory courses or buy additional study resources, and that they may experience less study time as they may work longer hour to financially support themselves through college.

The relationship between family income and MCAT scores has been widely described since 1995 [21]. Despite the MCAT exam being revised in 2015, recent studies have re-demonstrated the persistence of differences in scores relative to income groups with the new scoring scale [22]. Analysis by Terregino et al. reported that applicants with MCAT scores in the middle-third of the score scale were more likely to be UiM, FG college students, or from lower parental educational backgrounds than applicants from the upper-third of the score scale [23]. Additionally, their study found that admissions committees that accepted applicants with MCAT scores in the middle-third of the scale selected more diverse medical school classes.

Multivariable logistic regression found that UiM identity had a very strong positive association with acceptance and an odds ratio that was orders of magnitude larger than that of first-generation student status. The strength of the predictor is likely a reflection of adherence to critical and beneficial guidelines for diversity and inclusion in medical school admissions [5]. Although this admission practice has not yet resulted in significantly increased representation of UiM students in medicine compared to the US population, our results suggest that institutions are at least making deliberate efforts to consider students from these backgrounds [2, 24]. Extending these intentional efforts towards FG and LI applicants is encouraged. The data are there: AMCAS includes a first-generation college student indicator and fields for students to indicate and describe a disadvantaged background [6, 7, 25, 26]. Concurrent with admission committees using the available data in holistic review, activities such as pipeline program support, funded test-prep and application resources, and targeted outreach to and recruitment of socioeconomically disadvantaged (SED) college students may help increase their representation. These activities targeting UiM and SED individuals prior to medical school applications may increase the number of applicants from these groups. Notably, these individuals were underrepresented in the applicant pool and thus suggest that barriers may exist earlier in life and thus further limit the ability to diversify the medical profession.

While our study’s strengths include reporting on a large, standardized dataset across multiple admissions cycles, there are limitations. Use of the AMCAS data file precluded inclusion of applicants who applied solely to US osteopathic medicine programs. Furthermore, this study did not assess other data points that are important parts of the application process such as applicant’s essays, or interviews, which may contribute to differences observed in acceptance rates among the groups in this study. Notably, this study did not include variables such as age or applicant-reported sex in multivariable analyses as they did not have a statistically significant difference in univariable analyses. We were also unable to fine-tune the income data by controlling for factors such as family size and geographic income averages. Similarly, we did not have access to the time frame or number of hours dedicated to each extracurricular activity. Furthermore, this large cohort study was unable to assess the impact of different admission practices and missions at individual medical schools. Finally, we recognize that a plurality of medical schools incorporates a “secondary application” which may further influence a committee’s holistic evaluation and candidacy for acceptance. Future studies using similar data but with a medical school indicator may help highlight exemplary schools.

## Conclusions

The findings in this study suggest that medical school admission practices are commensurate with holistic review as acceptance to medical schools is positively associated with participation in all kinds of extracurricular activities, high MCAT score, high GPA, and UiM background when controlling for MCAT and GPA. While this study was unable to evaluate the differences in outcomes based on individual medical schools’ missions and admission practices, it is possible that this overreliance on science GPA and MCAT for medical school acceptance negatively impacts the diversity and representation of UiM and SED applicants. On the other hand, some strides have been made in the likelihood of acceptance for UiM applicants, albeit given high GPAs and MCAT scores. To eventually achieve a diverse US physician workforce, admission committees will need an ongoing commitment to practicing holistic applicant assessment. Additionally, applicants from UiM and socioeconomically disadvantaged backgrounds may benefit from greater academic support in high school and college and in preparation for the MCAT. Finally, FG and LI students may not have the same opportunities for engagement in extracurricular activities, possibly impacted by their financial responsibilities, which may be suggested by the data showing a higher level of participation in employment activities. However, if able to participate in extracurricular activities, this does have a modest impact on the likelihood of being accepted to medical school.

### Supplementary Information

**Additional file 1:** **Figure S1.** Relationshihp Between FG, LI, UiM, MCAT Score, GPA, and activities for applicants regardless of acceptance decision.

## Data Availability

The data used in this project were originally collected by the American Medical College Application Service (AMCAS) and maintained within the AAMC Applicant Matriculant Data file. Data were obtained from the AAMC through the standard data request process. Others wishing to use the data would need to file a request by emailing datarequest@aamc.org. This material is based upon data provided by the Association of American Medical Colleges (“AAMC”). The views expressed herein are those of the authors and do not necessarily reflect the position or policy of the AAMC.

## Abbreviations

US: United States
UiM: Underrepresented in medicine
GPA: Grade point average
MCAT: Medical college admissions test
FG: First-generation
LI: Low-income
LCME: Liaison committee for medical education
AAMC: American association of medical colleges
AMCAS: American medical college application service
FAP: Fee assistance program
